# Novel flow cytometric approach for the detection of adipocyte subpopulations during adipogenesis[S]

**DOI:** 10.1194/jlr.D065664

**Published:** 2016-04

**Authors:** Chrisna Durandt, Fiona A. van Vollenstee, Carla Dessels, Karlien Kallmeyer, Danielle de Villiers, Candice Murdoch, Marnie Potgieter, Michael S. Pepper

**Affiliations:** Institute for Cellular and Molecular Medicine, South African Medical Research Council Extramural Unit for Stem Cell Research and Therapy, Department of Immunology, Faculty of Health Sciences, University of Pretoria, Pretoria, South Africa

**Keywords:** adipocyte differentiation, adipose tissue-derived stromal cells, Bodipy 493/503, cluster of differentiation 36, fatty/acid binding protein, gene expression, lipid droplet, Nile Red, triglyceride

## Abstract

The ability of mesenchymal stromal cells (MSCs) to differentiate into adipocytes provides a cellular model of human origin to study adipogenesis in vitro. One of the major challenges in studying adipogenesis is the lack of tools to identify and monitor the differentiation of various subpopulations within the heterogeneous pool of MSCs. Cluster of differentiation (CD)36 plays an important role in the formation of intracellular lipid droplets, a key characteristic of adipocyte differentiation/maturation. The objective of this study was to develop a reproducible quantitative method to study adipocyte differentiation by comparing two lipophilic dyes [Nile Red (NR) and Bodipy 493/503] in combination with CD36 surface marker staining. We identified a subpopulation of adipose-derived stromal cells that express CD36 at intermediate/high levels and show that combining CD36 cell surface staining with neutral lipid-specific staining allows us to monitor differentiation of adipose-derived stromal cells that express CD36^intermediate/high^ during adipocyte differentiation in vitro. The gradual increase of CD36^intermediate/high/^NR^positive^ cells during the 21 day adipogenesis induction period correlated with upregulation of adipogenesis-associated gene expression.

Mesenchymal stromal cells (MSCs) are derived from the stroma of several tissues and contain a subpopulation of multipotent stem cells that have the ability to differentiate into a number of functional cell types, including osteoblasts, chondrocytes, and adipocytes (1–3). MSCs can be found in almost all tissues of the body and have been successfully isolated from bone marrow, adipose tissue, cord blood, and peripheral blood (1, 4, 5). MSCs are characterized in vitro by a minimum set of three criteria, as proposed by the Mesenchymal and Tissue Stem Cell Committee of the International Society of Cellular Therapy. According to these criteria, MSCs should be: *i*) plastic adherent; *ii*) express a defined set of surface antigens; and *iii*) be able to differentiate into osteoblasts, adipocytes, and chondrocytes in vitro (3, 6). The International Fat Applied Technology Society recommended that adipose-derived cells that meet the above-mentioned criteria should be referred to as adipose-derived stromal cells (ASCs) (3, 7, 8).

Adipocytes are no longer seen as being exclusively lipid- and energy-storing cells, but are viewed as complex endocrine cells that play an important role in body homeostasis (9–11). Several cytokines, chemokines, hormones, and other factors are secreted by adipocytes (11–13), linking these cells to processes such as inflammation, angiogenesis, and metabolic disorders (10, 14–17).

Several studies have shown that preadipocytes and mature adipocytes have different functions during adipogenesis (11, 12, 18–21). Distinct differentiation-dependent differences have been observed between these two cell types in their cytokine/chemokine expression profiles (18, 20, 21). Adipocyte toll-like receptor expression profiles are also highly dependent on their differentiation state (preadipocytes vs. mature adipocytes) (10, 19, 20). In addition, the ratio of preadipocytes to mature adipocytes changes during inflammation (10, 22). A current limiting factor in fully understanding the different stages of adipogenesis is the inability to accurately define and study intermediate adipocyte subpopulations that exist between MSCs and mature adipocytes (23). In addition, the majority of adipogenesis studies are performed in vitro using murine cell culture models (24). During these studies, the involvement of two adipocyte populations, preadipocytes and mature adipocytes, has mainly been investigated. It is also important to recognize that adipose tissue distribution in rodents and humans is different (25). Therefore, translating observations made on rodent cell culture models to humans should be undertaken with caution. Primary human preadipocyte cultures may be more representative of adipogenesis in humans, and the ability to differentiate MSCs into adipocytes in vitro serves as a proxy for the study of adipogenesis in vivo. Development and improvement of techniques that more accurately represent adipocyte subpopulations during adipogenesis may assist in a better understanding of the adipogenesis process, as well as how other processes, like inflammation, influence the repertoire of the various cell populations present in adipose tissue.

Adipocyte differentiation is associated with an increase in intracellular lipid droplets (26–28). These lipid droplets are highly organized organelles, consisting of a neutral lipid (triglycerides and cholesterol esters) core and an outer phospholipid layer with various regulatory proteins embedded in it (29, 30). At a cellular level, adipocyte differentiation is commonly visualized using microscopy techniques after staining the cells with lipid-specific stains [Oil Red O, Nile Red (NR), Bodipy 493/503 (BDP)] (31–34). However, flow cytometry is increasingly being used as a technique to quantify adipocyte differentiation (33–37).

Cluster of differentiation (CD)36 is a multifunctional transmembrane protein that is highly expressed on the adipocyte cell surface (38) as well as various other cell types, including monocytes, macrophages, endothelial cells, and platelets (38–41). One of the main functions of CD36 is to serve as a fatty acid translocase by facilitating the uptake of free long-chain fatty acids into cells (38–41). The uptake of these free fatty acids is followed by esterification into triglycerides, which are then stored within cytosolic lipid droplets (42).

Adipogenesis is a complex multi-step process that involves various transcription factors in a well-described sequential manner. The process of adipogenesis begins with the up- and downregulation of various transcription factors during the clonal expansion phase. These transcription factors in turn activate other transcription factors, such as CCAAT/enhancer binding protein (C/EBP)α and PPARγ. The upregulation of C/EBPα and PPARγ are essential during the differentiation stage of adipogenesis. The latter stages of differentiation are accompanied by the activation of various genes associated with adipocyte maturation, including fatty acid binding proteins (FABPs) such as FABP4 (43–45).

BDP is a nonpolar lipid stain that has the ability to stain neutral lipids as well as other nonpolar oils and lipids. BDP emits green fluorescence when dissolved in these lipids/oils (product insert, https://www.lifetechnologies.com) (46, 47). In contrast, the emission spectrum of the lipophilic dye, NR, changes according to the lipid environment. NR emits yellow-gold fluorescence (emission wavelength >528 nm) when dissolved in neutral lipids, while it fluoresces in the deep-red spectrum (emission wavelength >590 nm) when dissolved in amphipathic lipids (lipids with polar as well as nonpolar regions) (48–51).

In this study, we combined a lipid-specific stain with surface marker (CD36) staining to monitor differentiation of ASCs of human origin into adipocytes in vitro. Two fluorescent lipid-specific stains were compared, namely, NR and BDP. By making use of the unique fluorescent emission profiles of these lipid-specific dyes, we show that by applying multi-parameter flow cytometry, we are able to distinguish between different cellular stages during adipocyte differentiation. We also demonstrate that adipocyte CD36 surface expression precedes the expected increase in intracellular lipid content associated with adipocyte differentiation. In addition, we demonstrate a linear relationship between the upregulation of the adipogenesis end-stage gene, FABP4, and the increase in CD36 surface expression during adipocyte differentiation/maturation.

## MATERIALS AND METHODS

### Materials

Collagenase type I, penicillin/streptomycin (Pen/Strep) broad spectrum antibiotic cocktail, trypsin-EDTA (0.25%), PBS, α-MEM, and DMEM culture media were purchased from Gibco/Invitrogen (Carlsbad, CA). VersaLyse was purchased from Beckman Coulter (Miami, FL). FBS was purchased from Lonza (Basel, Switzerland). Dexamethasone, 3-isobutyl-methylxanthine, indomethacin, and human insulin were purchased from Sigma-Aldrich (St. Louis, MO). The 4′,6-diamino-2-phenylindole dihydrochloride (DAPI), Vybrant DyeCycle Ruby, NR, and BDP were purchased from Thermo Fisher Scientific/Life Technologies (Eugene, OR). Mouse anti-human CD36 conjugated to the fluorochrome, allophycocyanin (APC) (clone 5-271), was purchased from Biolegend (San Diego, CA).

### Isolation of ASCs from adipose tissue

ASCs were isolated, with minor modifications, from human adipose tissue as previously described (52, 53). Subcutaneous adipose tissue was obtained from 10 healthy donors that underwent elective liposuction procedures under general anesthesia. Informed consent was obtained from all donors. The study was approved by the Ethics Committee, Faculty of Health Sciences, University of Pretoria, study numbers 218/2010 and 421/2013. Briefly, excess oil was removed by washing the harvested adipose tissue in PBS. Adipose tissue was digested by constant agitation for 45 min at 37°C using 0.1% collagenase type I prepared in PBS supplemented with 2% Pen/Strep. The adipose-derived stromal vascular fraction was separated from debris and connective tissue by centrifugation (400 *g*, 5 min). Pellets were resuspended in VersaLyse and incubated for 10 min at room temperature to lyse contaminating red blood cells. After centrifugation, the cells were resuspended in culture medium. The culture medium consisted of α-MEM supplemented with 10% FBS and 2% Pen/Strep. The cell suspension was filtered through a 70 micron Falcon cell strainer (BD Biosciences, San Jose, CA) and was seeded (37°C, 5% CO_2_) at a density of 5 × 10^3^ cells/cm^2^ in 25 cm^2^ Nunc culture flasks (Nunc, Rosklide, Denmark). After 24 h, the flasks were rinsed with PBS to remove nonadherent cells, followed by the addition of fresh expansion medium.

### Expansion of ASCs

The plated cells were maintained at 37°C/5% CO_2_ in culture medium. At 80–90% confluence, cells were trypsinized for 10 min at 37°C using 0.25% trypsin-EDTA. Cells were replated at a density of 5 × 10^3^ cells/cm^2^ and cultures were expanded for 6 to 10 passages. All cultures were phenotyped at each passage. The cells used in differentiation experiments were positive for CD73, CD90, and CD105 and negative for CD34 and CD45.

### Adipocyte quantification using flow cytometry

A DAPI working solution was prepared in staining buffer [100 mM Tris (pH 7.4), 150 mM NaCl, 1 mM CaCl_2_, 0.5 mM MgCl_2_] to achieve a concentration of 10 μg/ml. NR and BDP working solutions were prepared in absolute ethanol to achieve concentrations of 2 μg/ml and 20 μg/ml, respectively. Prior to staining, the cells were trypsinized and washed using PBS supplemented with 10% FBS and 2% Pen/Strep. After centrifugation (184 *g*, 5 min), the pellets were resuspended in supplemented PBS and then stained with NR (20 ng/ml final concentration) or BDP (200 ng/ml), mouse anti-human CD36-APC, and DAPI (5 μg/ml final concentration). After 20 min incubation at room temperature, cells were analyzed using a Gallios flow cytometer (Beckman Coulter, Miami, FL). DAPI was excited with a 405 nm laser and fluorescence emissions were collected using the fluorescence detector (FL)9 [450/40 nm band pass filter (BP)] detector. NR was excited with a 488 nm laser and fluorescent emission signals were collected using FL2 (575/30 nm BP) and FL5 [755 nm long pass filter (LP)] detectors. Noninduced ASCs (day 1, 24 h post adipogenic induction) were used to optimize the photomultiplier tube voltage settings, as well as to set the signal-to-background threshold of the NR and BDP fluorescence. Compensation settings were optimized using a differentiated culture (day 21) by removing any fluorescent spill over into the FL2 and FL5 channels. After initial optimization, all instrument settings were kept constant for the duration of the study (supplementary Fig. 2). Flow Check Pro (Beckman Coulter) fluorospheres were run daily to validate instrument performance. Flow cytometry data were analyzed using Kaluza flow cytometry data analysis software (Version 1.3; Beckman Coulter).

### Fluorescence microscopy and analysis

Cells (both noninduced and induced) were cultured in 6-well plates as described above. Prior to fluorescence microscopy imaging, culture medium was removed and the wells were rinsed with PBS to remove nonadherent cells. PBS supplemented with 10% FBS and 2% Pen/Strep was added to the wells. Both noninduced and induced cultures were stained with 2.5 μg/ml (final concentration) DAPI. The cultures were incubated overnight in a 5% CO_2_ incubator to allow optimal staining of all nuclei. The next day cultures were stained with either NR (final concentration 50 ng/ml) or BDP (final concentration 500 ng/ml). Fluorescence images (20× objective; 256 × 256 pixels) were captured after a 20 min incubation at room temperature, using an AxioVert A1 inverted fluorescence microscope (Carl Zeiss, Gottingen, Germany) equipped with an AxioCam Cm1 camera (Carl Zeiss). Single channel images were captured and subsequently converted into overlay images. For NR, three single channel images were captured. The first single channel image was captured using Filter Set 9 (excitation BP 450–490, emission LP 515; Carl Zeiss) to visualize yellow-gold fluorescence. A second single channel image was captured using Filter Set 00 (excitation BP 530–585, emission LP 615; Carl Zeiss) to visualize deep-red fluorescence emission of lipid droplets. A third single image was captured using Filter Set 49 (excitation G 365, emission BP 445/50; Carl Zeiss) to visualize nuclei stained with DAPI. For BDP, two single color images were captured using Filter Set 9 and Filter Set 49, respectively. Images were initially captured using AxioVision software (Version 4.8.2).

In order to optimally visualize lipid droplets, all images were enhanced, but not manipulated, post-acquisition using Image J imaging software (54). Enhancement of images was done by adjusting contrast and brightness settings.

### RNA isolation and RT-quantitative PCR

Total RNA was extracted from postconfluent ASCs using the RNeasy mini kit (Qiagen, Hilden, Germany) according to the manufacturer’s instructions. cDNA was generated using the iScript™ reverse transcription supermix (Bio-Rad Laboratories, Inc., Hercules, CA). For RT-quantitative (q)PCR, LightCycler^®^ 480 SYBR Green I Master Mix (Roche, Basel, Switzerland) was used. PCR reactions were performed in 10 μl volumes, where the primer concentrations were 400 nM and the cDNA concentration was 20 ng/μl. qPCR was performed on a LightCycler 480 II instrument (Roche) using the following conditions: denaturation at 95°C for 5 min, 45 cycles of amplification at 95°C for 30 s, 62°C for 30 s, 72°C for 30 s. After amplification, a melt curve was performed at 95°C for 30 s, 40°C for 30 s, and ramped at 0.1°C/s. The primers (IDT, Coralville, IA) for the genes of interest and the reference genes (internal controls) are indicated in **Table 1**.

**TABLE 1.** Primer pairs

Gene	Forward (3′ to 5′)	Reverse (3′ to 5′)
Genes of interest		
Preadipocyte factor 1 (*Pref-1*)	ACTGTGGGTATCGTCTTCCT	AGCAGCAGGTTCTTCTTCTTC
*C/EBPβ*	GACAAGCACAGCGACGAGTA	AGCTGCTCCACCTTCTTCTG
*C/EBPα*	GTCTCTGCTAAACCACCA	AAAGGAAAGGGAGTCTCAG
*PPARγ*	CGTGGATCTCTCCGTAAT	TGGATCTGTTCTTGTGAATG
*FABP4*	GCTTTGCCACCAGGAAAGTG	ATGACGCATTCCACCACCAG
Reference genes		
*GUSB*	GATCGCTCACACCAAATC	TCGTGATACCAAGAGTAGTAG
*PPIA*	GAGTTAAGAGTGTTGATGTAGG	CCTGGGACTGGAAAGTAA
*TBP*	CCGAAACGCCGAATATAA	GGACTGTTCTTCACTCTTG
*YWHAZ*	TGACATTGGGTAGCATTAAC	GCACCTGACAAATAGAAAGA

### Statistical analysis

Results are expressed as means ± SD. Outliers were identified using the ROUT statistical test and excluded from statistical analysis. The nonparametric Mann-Whitney test was used to determine statistical significance between groups. The degree of correlation between different groups was performed using the nonparametric Spearman rank correlation test. GraphPad PRISM 6 (Version 6.07) and INSTAT software Version 3.06 (GraphPad Software Inc., La Jolla, CA) were used for all statistical analyses.

For the qPCR data, relative gene expression was calculated using the comparative C_T_ method. Relative fold-increase in gene expression (ΔC_T_) was reported as an increase in gene expression relative to the following housekeeping genes: glucuronidase β (GUSB), peptidylprolyl isomerase A (PPIA), TATA binding protein (TBP), and tyrosine 3-monooxygenase/tryptophan 5-monooxygenase activation protein zeta (YWHAZ) (Table 1).

Differences between groups were considered significant if the *P* values were ≤0.05, with *, **, and *** corresponding to *P* < 0.05, *P* < 0.01, and *P* < 0.001, respectively.

## RESULTS

Adipocytes are fragile cells (55, 56). During flow cytometric analysis, intact cells were identified according to their intermediate/high forward scatter characteristics (see supplementary Fig. 1). To confirm that these cells (intermediate/high forward scatter) were viable, the cells were stained with the nuclear stain, DAPI (5 μM, final concentration). The viability of the cells identified as intact was 96.57 ± 3.33% (noninduced) and 93.87 ± 8.5% (induced), respectively. Our results confirm previous findings that larger mature adipocytes seem to be more fragile and susceptible to damage during sample processing and analysis (55).

**Fig. 1.**
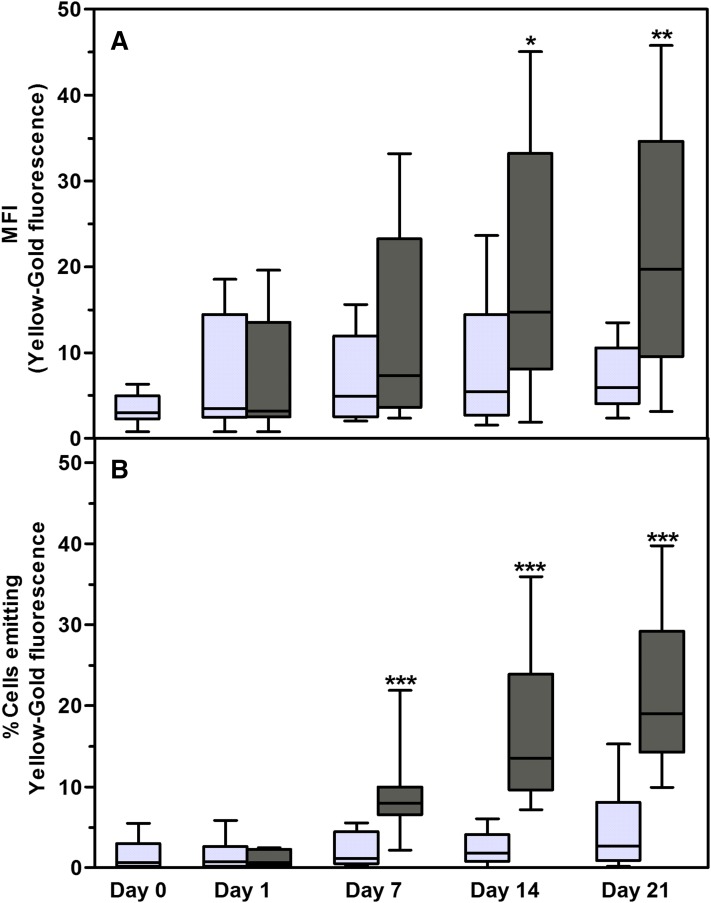
MFI (A) as well as percentage (B) of intact cells that emit yellow-gold fluorescence (515–565 nm) after staining with NR. Cultures were terminated on days 0 (n = 10), 1 (n = 9), 7 (n = 13), 14 (n = 13), and 21 (n = 13) after induction of adipogenesis. Results are displayed as minimum/maximum box-whisker plots where the median value is indicated by the solid horizontal line within each box. ASC cultures were from 10 different donors. Results from noninduced cultures are indicated by light gray box-whisker boxes; results from differentiated cells (adipocytes) are indicated by dark gray boxes. *P < 0.5; **P < 0.01; ***P < 0.001.

**Fig. 2.**
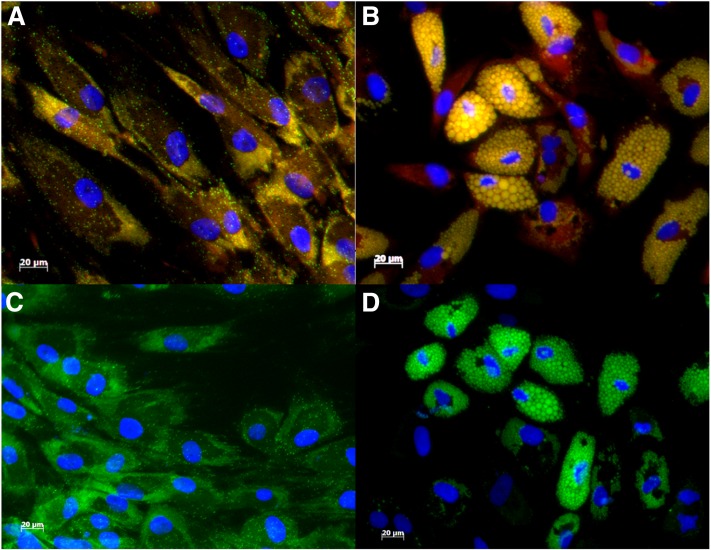
Fluorescence microscopy images of noninduced ASCs (A, C) and differentiated adipocytes (B, D) after staining with NR (A, B) and BDP (C, D). DAPI was used to visualize the nuclei. Images were obtained 21 days post induction. Images were initially captured as single channel images and then merged using Image J software.

The first indication of adipocyte differentiation is the appearance of lipid droplets in the cytoplasm of differentiating preadipocytes (49). The lipid droplet core consists mainly of neutral lipids, triglycerides, and cholesterol esters (27, 57). The lipophilic dye, NR, emits yellow-gold (emission wavelength >528 nm) fluorescence when dissolved in neutral lipids (48, 50). Adipocytes were stained with NR at various time points (days 0, 1, 7, 14, and 21) after adipocyte differentiation was induced. The percentage of cells that emitted yellow-gold fluorescence due to the formation of intracellular lipid droplets increased gradually over the 21 day period (**Fig. 1B**). On day 7, 8.57 ± 5.05% (*P* < 0.0001 compared with day 1) intact cells emitted yellow-gold fluorescence. This increased to 16.66 ± 8.88% on day 14 (*P* < 0.0001 compared with day 1). By day 21, the percentage of intact cells that emitted yellow-gold fluorescence had further increased to 21.46 ± 9.44% (*P* < 0.0001 compared with day 1). Initially, approximately 1% of cells in the noninduced cultures emitted low levels of yellow-gold fluorescence (Fig. 1B). By day 21, the proportion of nondifferentiated ASCs in noninduced cultures that emitted low levels of yellow-gold fluorescence had increased to 4.70 ± 5.20% (Fig. 1B). This observed increase in the percentage of noninduced cells that emitted low levels of yellow-gold fluorescence was not statistically significant and may have contributed to the high levels of variance observed between the ASC cultures that were investigated. The noninduced cultures were confluent on day 21, resulting in contact inhibition. The observed increase in yellow-gold fluorescence emitted by these nondifferentiated ASCs is consistent with previous findings that lipid droplet biogenesis occurs in cells under stress, i.e., during contact inhibition (58, 59). Our results support previous findings (60) that lipid droplets present in cells other than adipocytes are usually very small in size (<1 μm in diameter) (**Fig. 2A, C**), compared with the intracellular lipid droplets that form during adipocyte differentiation (Fig. 2B, D). A gradual increase in the median fluorescence intensity (MFI) was observed in the induced cultures (Fig. 1A).The median yellow-gold fluorescence intensities observed in induced ASCs were significantly higher at day 14 (*P* = 0.016) and day 21 (*P* < 0.008) when compared with the MFIs observed for the noninduced cells at the corresponding time points. No changes were observed in the median yellow-gold fluorescence intensities observed for the noninduced cultures during the 21 day culture period (Fig. 1A).

**Fig. 3.**
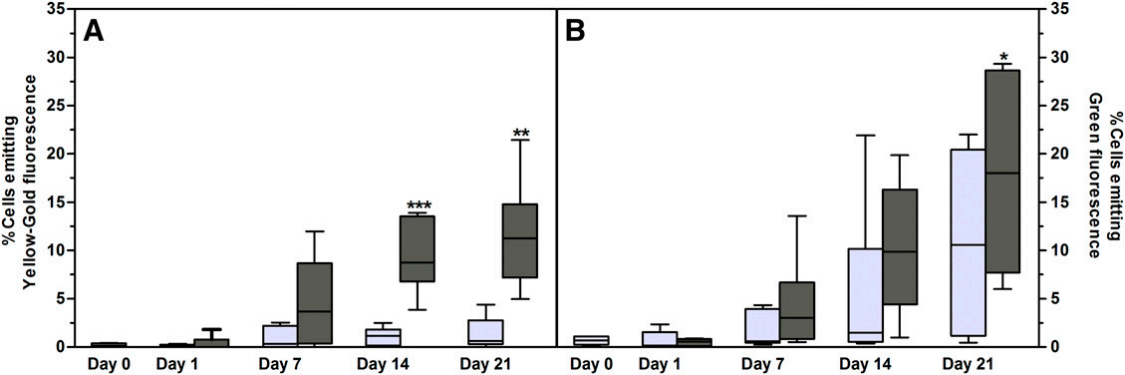
A comparison of NR and BDP detection of lipid droplets in ASC cultures from seven independent donors. Cultures were terminated on days 0, 1, 7, 14, and 21 after induction of adipogenesis. A: Percentage of intact cells that emit yellow-gold fluorescence (515–565 nm) after staining with NR. B: Percentage of intact cells that emit green fluorescence (515–535 nm) after staining with BDP. The positive detection limits were set according to auto-fluorescence levels detected at day 0. Results are displayed as minimum/maximum box-whisker plots where the median value is indicated by the solid horizontal line within each box. Results from noninduced cultures are indicated by light gray box-whisker boxes; results from differentiated cells (adipocytes) are indicated by dark gray boxes. Asterisks (*) indicate statistical significance at the specific time point when compared with the same culture condition at day 1. *P < 0.5; **P < 0.01; ***P < 0.001.

**Fig. 4.**
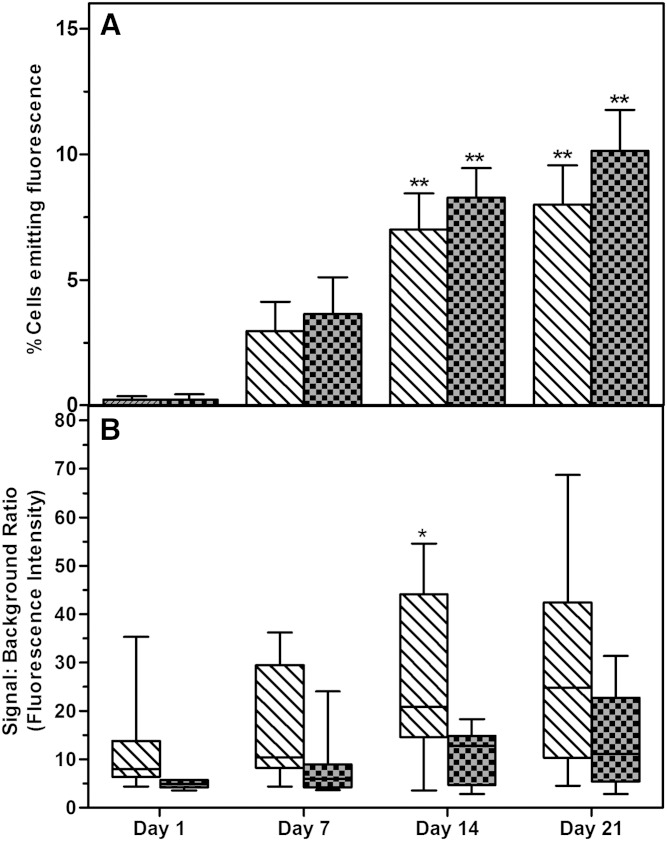
Comparison of intracellular neutral lipid accumulation after staining with NR and BDP, respectively, in ASCs induced to differentiate into adipocytes. A: Percentage of intact cells from induced cultures that emit neutral lipid-specific fluorescence. Results are expressed as mean percent of cells emitting lipid-specific fluorescence ± SD. B: The signal (emitted fluorescence):background fluorescence ratio after staining with NR and BDP. Signal:background ratio results are displayed as minimum/maximum box-whisker plots where the median value is indicated by the solid horizontal line within each box. The positive detection limits (region) were adjusted to compensate for the noninduced associated changes in fluorescence. The bars with diagonal fill indicate the results (corrected) obtained for cultures stained with NR. The bars with checker box fill indicate the results (corrected) obtained for cultures stained with BDP. Asterisks (*) indicate statistical significance at the specific time point when compared with the same staining condition at day 1. *P < 0.5; **P < 0.01.

A second neutral lipid-specific dye was included, namely, BDP. The sensitivity of NR and BDP to detect, using flow cytometry, intracellular lipid droplets during adipogenesis was directly compared in ASC cultures from seven independent donors (**Fig. 3**). BDP is a new class of lipid-specific fluorescent dyes and has the capacity to stain neutral and other nonpolar lipids. It is described as being more sensitive than NR for staining lipid droplets (61). In general, a greater proportion of cells emitted fluorescence associated with increased neutral lipid content when stained with BDP compared with NR (Fig. 3A, B). Day 1 cultures (noninduced and induced, respectively) were used to set the positive detection limits for both NR and BDP (Fig. 3A, B). Although the differences that were observed between NR and BDP were not statistically significant, NR staining resulted in a significantly higher proportion of cells that emitted yellow-gold fluorescence post induction when compared with the noninduced cells at day 7 (*P* = 0.036), day 14 (*P* = 0.0006), and day 21 (*P* = 0.0006). A gradual increase in fluorescence was observed over the 21 day period when the noninduced cultures were stained with BDP, resulting in nonsignificant differences between the noninduced and induced cultures (Fig. 3B). In order to compare the proportion of cells with increased neutral lipid content associated with adipogenesis as detected by NR and BDP, the detection limits were adjusted to compensate for the increase in fluorescence observed in the noninduced cultures (**Fig. 4**). When the detection limits were set according to each time point’s control (noninduced cultures), both NR and BDP detected a similar proportion of cells (Fig. 4A; *r*^2^ = 1.0; *P* = 0.08). In order to compare the relative fluorescence intensities of NR and BDP, the signal (emitted fluorescence) to background fluorescence ratios was calculated using the following formula: signal:background ratio = MFI of cells emitting fluorescence/MFI of cells only emitting background fluorescence. Although variable, we found that NR provided a better resolution between cells emitting fluorescence and cells that did not emit fluorescence (signal:background ratio) when compared with the signal:background ratio observed for BDP (Fig. 4B; supplementary Fig. 2).

**Fig. 5.**
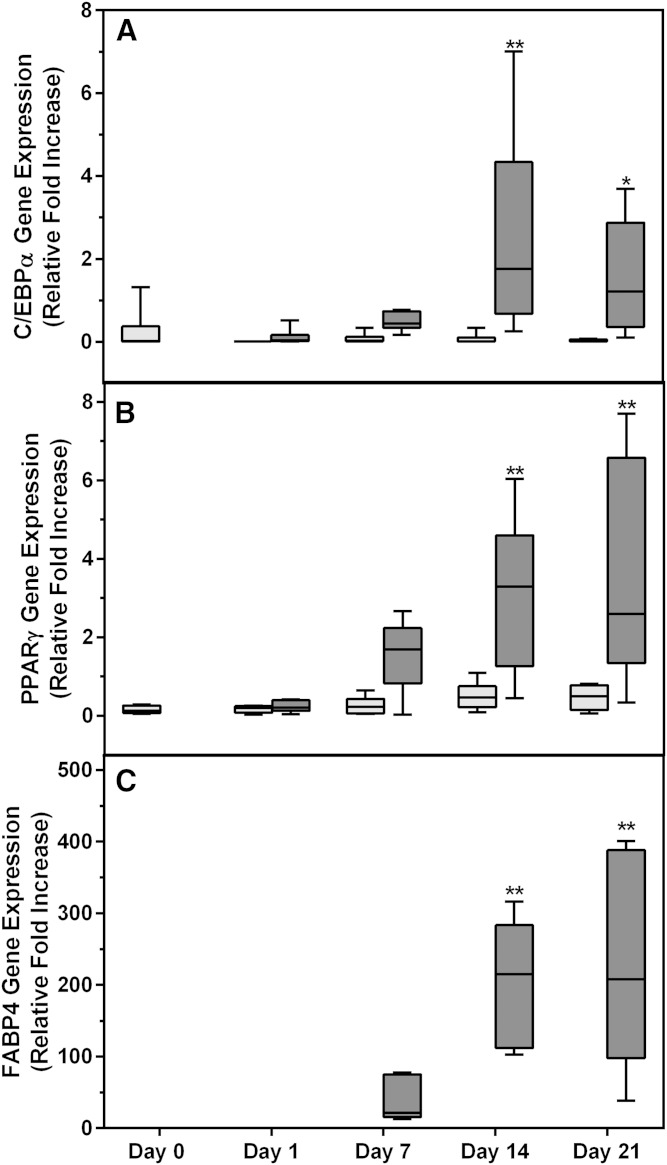
Levels of expression of genes associated with adipocyte differentiation. C/EBPα (A); PPARγ (B); FABP4 (C). Cultures were terminated on days 0, 1, 7, 14, and 21, respectively, after induction of adipogenesis. Relative fold-increase in gene expression (ΔC_T_) was reported as fold-increase of gene expression relative to the following housekeeping genes: GUSB, PPIA, TBP, and YWHAZ. All results are expressed as mean ± SD from six ASC cultures. Noninduced cultures are indicated by light gray bars; differentiated cells (adipocytes) are indicated by dark gray bars. *P < 0.5; **P < 0.01.

In order to confirm that the increase in neutral lipid content observed in the noninduced samples was not due to the spontaneous differentiation of ASCs, gene expression studies were performed. C/EBPα and PPARγ are two of several transcription factors that play an important role in adipocyte differentiation. These transcription factors are involved in a well-described cascade of molecular events that leads to the expression of proteins, such as FABP4, that are characteristic of mature adipocytes (45, 62, 63). The baseline level of expression of these genes in ASCs is very low. The relative baseline gene expression of C/EBPα, PPARγ, and FABP4 in ASC cultures (n = 6) prior to adipogenic induction (day 0) was 0.24 ± 0.53, 0.16 ± 0.10, and 0.005 ± 0.005, respectively. None of these genes were significantly upregulated in noninduced ASC cultures during the 21 day culture period (**Fig. 5A–C**), confirming that the increase in lipid accumulation observed at day 21 is not due to spontaneous differentiation of the ASCs into adipocytes. In contrast, all three genes were significantly upregulated in the induced cultures (Fig. 5A–C). C/EBPα and PPARγ were significantly increased from day 14 onwards (Fig. 5A, B), while FABP4 was significantly upregulated from day 7 onwards (Fig. 5C). The relative fold-increase for FABP4 gene expression was very low (<0.15) in the noninduced cultures.

**Fig. 6.**
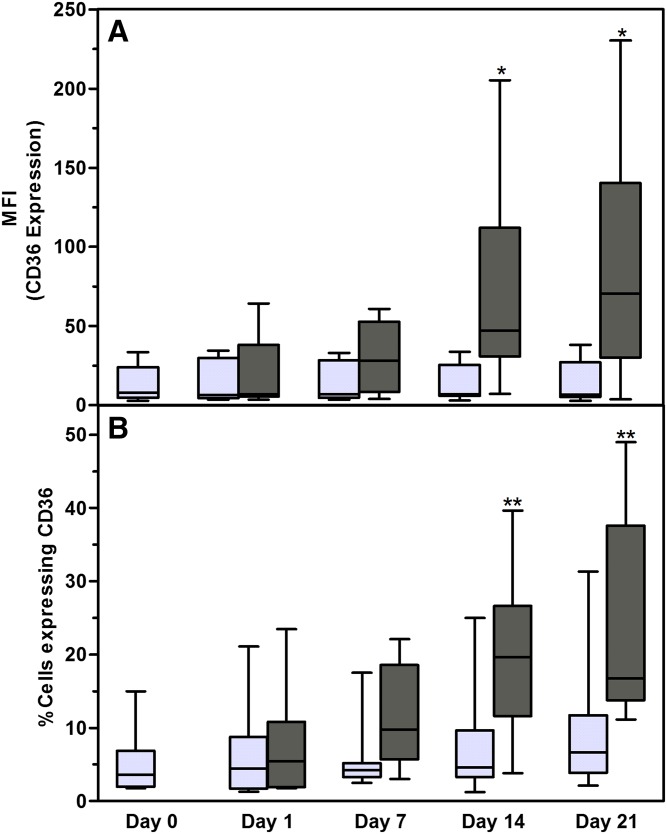
Percentage of intact cells that express CD36 at intermediate/high levels. Cultures were terminated on days 0, 1, 7, 14, and 21 after induction of adipogenesis. Results are displayed as minimum/maximum box-whisker plots where the median value is indicated by the solid horizontal line within each box. ASC cultures were from nine different donors. A: MFI of CD36^intermediate/high^-expressing cells. B: Percentage of cells with intermediate/high CD36 expression. Results represent nine ASC cultures. Results from noninduced cultures are indicated by light gray bars; results from differentiated cells (adipocytes) are indicated dark gray bars. *P < 0.5; **P < 0.01.

**Fig. 7.**
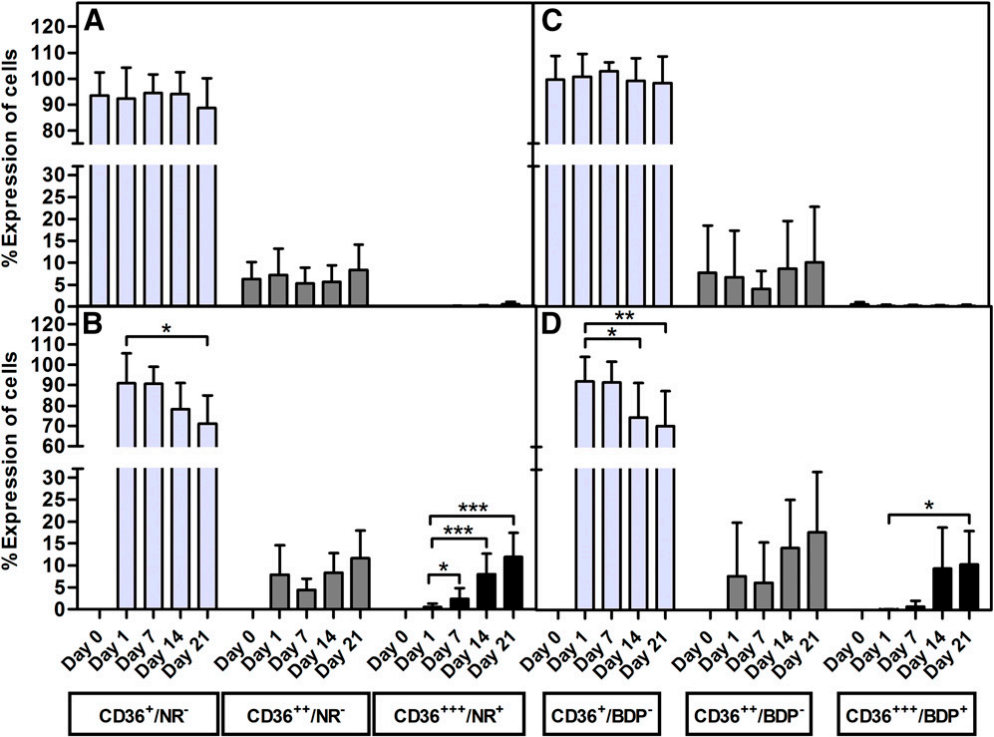
An increase in the surface expression of CD36 precedes an increase in intracellular neutral lipid content during adipocyte differentiation. Results are expressed as percent of intact cells emitting both neutral lipid-specific stain and CD36-associated fluorescence. Results represent the mean ± SD of eight independent donors’ ASC cultures. Cultures were simultaneously stained with mouse anti-human CD36-APC, as well as a lipid-specific fluorescent dye. A: Noninduced cells stained with CD36-APC and NR. B: Induced cells stained with CD36-APC and NR. C: Noninduced cells stained with CD36-APC and BDP. D: Induced cells stained with CD36-APC and BDP. Asterisks (*) indicate statistical significance at the specific time point when compared with the same subpopulation at day 1. *P < 0.5; *** *P* < 0.001.

Christiaens et al. (64) demonstrated, using the murine 3T3 cell line, that CD36 is directly involved in adipocyte differentiation and adipogenesis. In this study, we combined CD36 surface expression staining with a lipid-specific fluorescent stain to monitor adipocyte differentiation in vitro. According to the International Fat Applied Technology Society and the International Society of Cellular Therapy guidelines, ASCs should dimly express CD36 (3). In this study, 97.17 ± 4.92% (n = 10; MFI: 2.52 ± 2.14) of the ASCs expressed CD36 dimly. A subpopulation of ASCs (5.13 ± 4.22%) expressed CD36 at a higher intensity (MFI: 14.01 ± 11.52) (supplementary Fig. 3). These CD36^intermediate/high^ cells were the cells of interest in this study. The proportion of CD36^intermediate/high^ cells, as well as the expression level of CD36, increased gradually over the 21 day culture period when ASCs were induced to differentiate into adipocytes (**Fig. 6A, B**). At day 1 (24 h after induction was initiated) 10.4 ± 4.5% CD36^intermediate/high^-positive cells were observed. The proportion of the CD36^intermediate/high^-positive cells increased to 11.63 ± 7.03% on day 7 (*P* = 0.34 compared with day 1), 20.15 ± 10.86% on day 14 (*P* = 0.0099 compared with day 1), and 25.25 ± 13.95% on day 21 (*P* = 0.007 compared with day 1) (Fig. 6B). CD36 expression (as indicated by the MFI) was statistically significant on day 14 (*P* = 0.05) and day 21 (*P* = 0.05) when compared with the baseline level at day 0 (Fig. 6A). The observed increase in CD36^intermediate/high^ cells correlated (*r*^2^ = 1.0, *P* = 0.08, n = 4) with the proportion of cells with increased neutral lipid content. Our results also show that the upregulation of CD36 on the cell surface of adipocytes precedes an increase in intracellular lipid content (**Fig. 7**; supplementary Figs. 4, 5). From the flow cytometry data, it is clear that cells progress from expressing CD36 dimly (MFI: 5.09 ± 5.40) with no detectable increase in intracellular neutral lipid content (CD36^+^/BDP^−^ or NR^−^) to an increase in expression of CD36 (MFI: 59.14 ± 51.02), while the intracellular neutral lipid content remains undetectable (CD36^2+^/BDP^−^ or NR^−^) to cells that strongly express CD36 (MFI: 415.03 ± 289.24) with a detectable increase in the intracellular neutral lipid content (CD36^2+^/BDP^+^ or NR^+^) (Fig. 7; supplementary Figs. 4, 5; days 14 and 21).

**Fig. 8.**
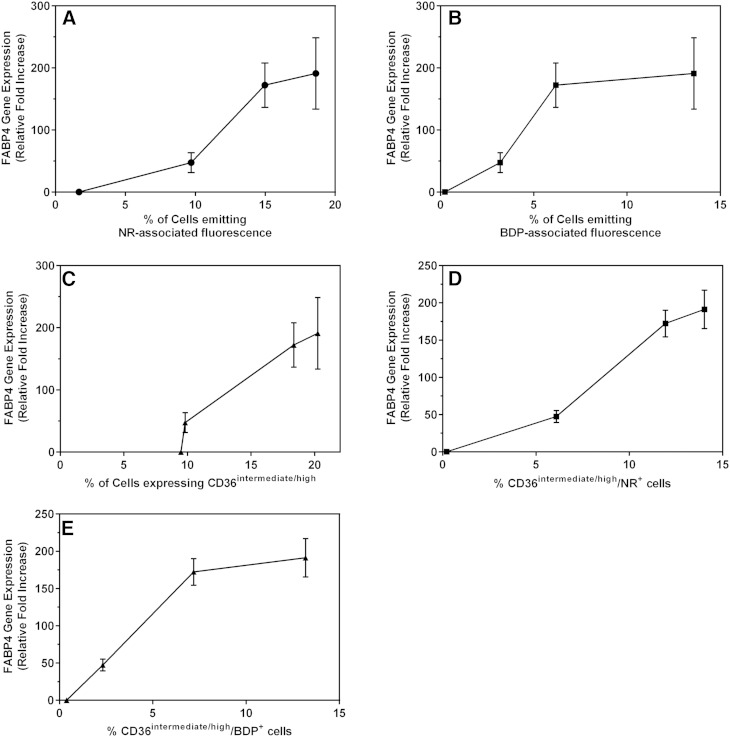
Correlation between FABP4 mRNA expression and mature adipocyte phenotypes as identified using flow cytometry. Correlation of FABP4 gene expression with an increase in intracellular neutral lipid content (total) after staining with NR (A); an increase in intracellular neutral lipid content (total) after staining with BDP (B); an increase in the intermediate/high expression levels of CD36 (C); the proportion of cells that highly express CD36 and simultaneously emitted yellow-gold fluorescence (NR-associated) (D); and proportion of cells that highly express CD36 and simultaneously emit green fluorescence after staining with BDP (E). Cultures were terminated on days 0, 1, 7, 14, and 21 after induction of adipogenesis. Gene expression levels were normalized to day 0. All results are expressed as mean ± SD and are from five independent ASC cultures from five different donors.

FABP4 is mainly expressed by mature adipocytes and macrophages (65). Similar to CD36, FABP4 binds long-chain fatty acids and, in so doing, facilitates their transport into cells, after which the fatty acids are esterified and stored as triglycerides within intracellular lipid droplets (42). The upregulation of FABP4 is associated with the latter stages of adipocyte differentiation, and is therefore an indication of the adipocyte maturation process. We investigated the relationship between FABP4 gene expression and CD36^intermediate/high^ expression, as well as FABP4 gene expression and intracellular lipid content, as measured by flow cytometry (**Fig. 8A–E**). FABP4 gene expression (relative fold increase; n = 6) correlated significantly with intracellular neutral lipid content as detected by both NR and BDP staining, as well as with CD36^intermedate/high^ expression in induced cultures over the 21 day culture period (Fig. 8A–E).

**Fig. 9.**
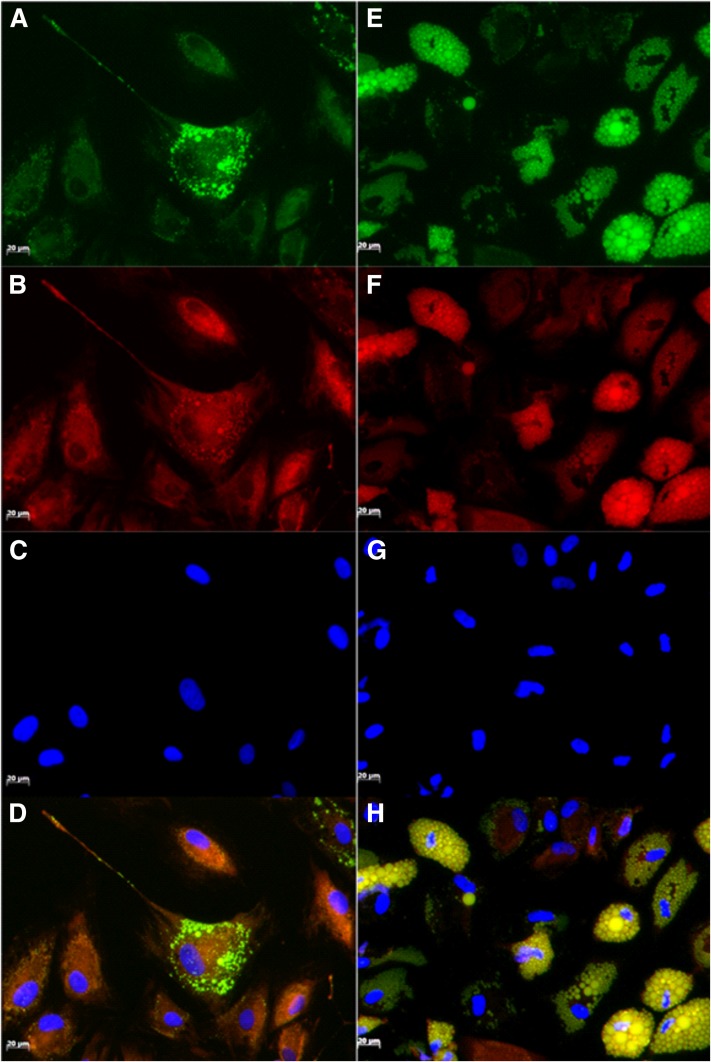
Fluorescence microscope images of ASCs at 7 days (A–D) and 21 days (E–H) after induction of adipogenesis. Cells were simultaneously stained with DAPI and NR. Images were captured as single channel images. Yellow-gold fluorescence lipid droplets were visualized using an excitation of 450–490 nm and an emission of 515–565 nm (A, E). Lipid droplets emitting deep-red fluorescence were visualized using an excitation of 530–585 nm and an emission of >615 nm (B, F). Nuclei were visualized using an excitation of 365 and an emission of 425–465 nm (C, G). Single channel images were merged using Image J software (D, H).

**Fig. 10.**
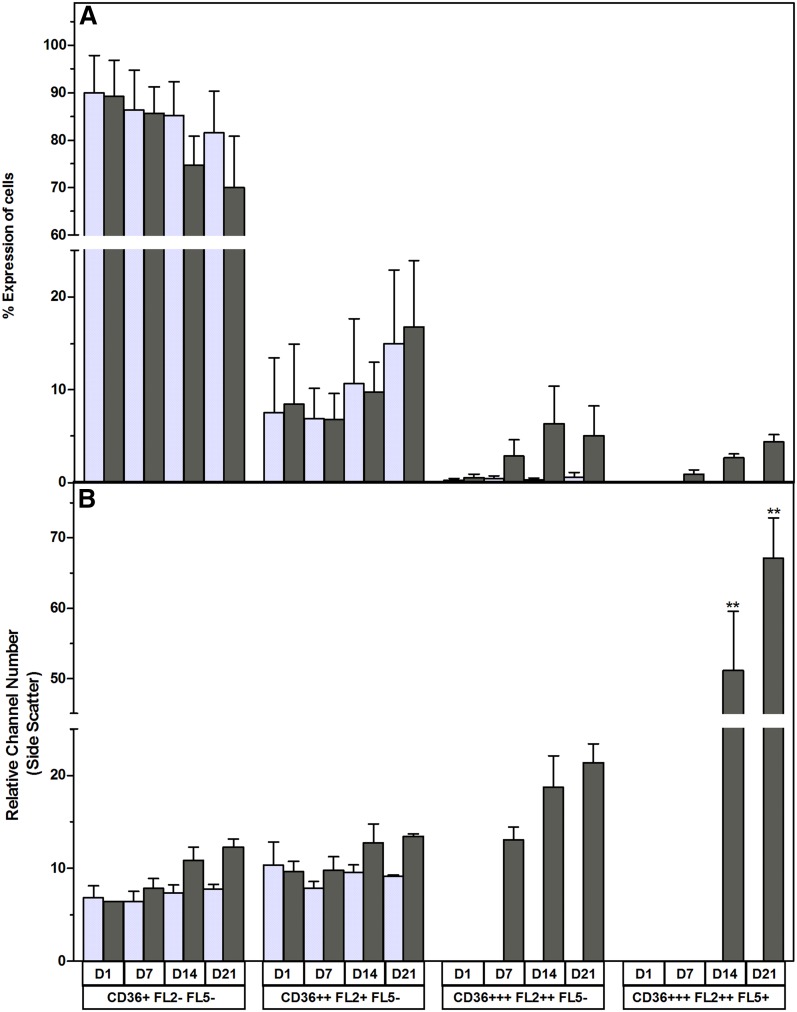
Maturation of adipocytes in vitro is associated with an increase in cellular complexity. Cells were stained with CD36 APC and NR. A: Percent expression. Results are expressed as the mean percent expression ± SD of three ASC cultures over a 21 day period. Noninduced cultures are indicated by light gray bars; differentiated cells (adipocytes) are indicated by dark gray bars. **P < 0.01.

The outer layers of lipid droplets consist of amphipathic lipids, mainly cholesterol and phospholipids. As preadipocytes differentiate into mature adipocytes, the lipid droplets merge to eventually form one large unilocular droplet that occupies around 90% of the cytoplasm (27, 49). Greenspan, Mayer, and Fowler (48) showed that NR emits deep-red fluorescence (emission wavelength >590 nm) when it binds to amphipathic lipids. We postulated that these unique emission properties of NR would allow us to identify a more mature adipocyte phenotype during the end-stages of adipocyte differentiation in vitro. The maturation of adipocytes is associated with an increase in the number, as well as in the size, of intracellular lipid droplets, which consequently contribute to an increase in amphipathic lipid quantities present in adipocytes (**Fig. 9A–H**). Our results show that an increase in amphipathic lipids results in the emission of deep-red fluorescence (FL5+) when stained with NR (**Fig. 10A**; supplementary Fig. 5; days 14 and 21). Due to the absence of relatively large lipid droplets in the noninduced cultures, these cultures did not emit fluorescence in the deep-red spectrum (emission wavelength >590 nm) when stained with NR (Fig. 10A; supplementary Fig. 5; days 14 and 21).

## DISCUSSION

One of the hallmarks of MSCs is their ability to differentiate into adipocytes in vitro (6). The histochemical stain, Oil Red O, is commonly used to visualize adipocytes during differentiation in vitro (1, 5, 33, 34, 49, 53, 60, 66–68). However, due to technical difficulties that are often encountered with Oil Red O staining, many investigators have sought alternative ways to monitor adipocyte differentiation. One alternative is the fluorescent lipophilic stain, NR (9-diethylamino-5H-benzo[α]phenoxazine-5-one), which is used to measure adipogenesis using fluorescence microscopy (33, 35, 49, 60, 66, 68). Recently, the fluorescent lipophilic stain, BDP (4,4-difluoro-1,3,5,7,8-pentamethyl-4-bora-3a,4a-diaza-s-indacene), has also been used to detect intracellular lipid droplets using microscopy (46, 69–72). It has been reported that BDP is more sensitive in detecting intracellular lipid droplets when compared with NR (61). Aldridge et al. (34) showed that a NR-based flow cytometric assay is more quantitative and less subjective than the Oil Red O assay. Lansdown, Ludgate, and Rees (73) also commented that either NR flow cytometry or qPCR should be the methods of choice for monitoring adipocyte differentiation. To our knowledge, this is the first study that has used BDP to monitor adipocyte differentiation using flow cytometry.

Currently, all NR-based flow cytometric studies make use of the ability of NR to emit yellow-gold fluorescence (emission between 505 and 585 nm) when dissolved in neutral lipids after excitation by a 488 nm light source (33, 35, 49, 60, 66, 68, 74). The emission of yellow-gold fluorescence is associated with the total neutral lipid content of cells when stained with NR. Lipid droplets are present in all cells, but are generally larger in size, as well as present in higher quantities, in adipocytes and macrophages. The core of these intracellular lipid droplets consists mainly of neutral lipids, triglycerides, and cholesterol esters (27, 57). Although NR has increasingly been used in flow cytometric assays to quantify adipocyte differentiation, there is no consensus in reporting flow cytometric results after staining adipocytes with NR. Most investigators report the flow cytometric results as both percentages of cells that emit yellow-gold fluorescence after staining with NR, as well as the mean or median fluorescence intensity of the emitted yellow-gold fluorescence. Other investigators choose to only report the mean or median fluorescence intensity of the emitted yellow-gold fluorescence. Our results confirm that yellow-gold median fluorescence intensity seems to be a more sensitive indication of adipocyte formation. We also observed that NR resulted in a better signal:background resolution compared with BDP (Fig. 4B). Our results indicate that BDP is more sensitive than NR in detecting changes in intracellular lipid content (Fig. 3B). The increase in sensitivity observed for BDP seems to result in the detection of nonadipogenesis-related changes in the neutral lipid content of the cells. This increase in intracellular lipid content observed in noninduced cells is not due to spontaneous differentiation of ASCs, as no upregulation of adipogenesis-associated transcription factors was observed.

Many investigators have shown that cell-to-cell contact leads to changes in the lipid content of cells. Thus, using murine 3T3 fibroblasts, it was observed that contact inhibition led to an increase in intramembraneous structures in these cells (75–77). The exact lipid composition of these intramembraneous structures is unknown, but interestingly, Cansell et al. (78) did not find any difference in the total phospholipid content between actively proliferating and confluent endothelial cells. It has also been reported that in addition to preventing proliferation, contact inhibition leads to changes in lipid droplet composition (78, 79). Cansell et al. (78) reported that contact inhibition led to an increase in intracellular cholesterol levels. Diaz et al. (79) suggest cell-to-cell contact changes the ratio of triglycerides to cholesterol esters present in the lipid droplet core by causing a decrease in the triglyceride content of the lipid. The fluorescence emission profile of NR seems to be sensitive to changes in the hydrophobic strengths of lipids (79). The predominant lipids present in the lipid droplet core are triglycerides, followed by cholesterol esters (69). A possible explanation for the differences in the detection sensitivity of NR and BDP may thus be due to the specificity of NR to emit yellow-gold fluorescence when dissolved in triglycerides. This results in NR being potentially more sensitive for adipogenesis-associated changes to the intracellular lipid content. BDP seems to be less specific to the hydrophobic strength of lipids and thus measures all neutral lipids irrespective of their hydrophobic strength. This hypothesis, however, needs to be tested directly in further studies.

CD36, a fatty acid translocase, plays an important role in fatty acid metabolism during lipid droplet formation (38–42). In this study, we show that by combining a lipid-specific stain with CD36 surface marker staining, we are able to identify three main subpopulations during adipocyte differentiation. The gene expression data confirm that both the increases in intracellular neutral lipid content, as well as the upregulation of CD36 cell surface expression, are associated with adipogenesis. PPARγ expression controls the expression of FABP4 during the later stages of adipocyte differentiation (26, 80). The exact function of FABPs, including FABP4, is not yet fully known, but one of the proposed functions of these proteins, also known as lipid chaperones, may be to facilitate the transport of lipids to specific compartments in the cell, such as to the lipid droplet for storage (80). CD36 is highly expressed by mature adipocytes (38, 81). The expression of CD36, therefore, may be used to monitor the transition from adipocyte differentiation to maturation, as we observed that an increase in CD36 expression precedes an increase in intracellular neutral lipid content. In addition, we observed an improved correlation of total neutral lipid content due to the intracellular accumulation of lipid droplets, with upregulation of FABP4, when the simultaneous detection of CD36^high^ expression and an increase in neutral lipid content was used (Fig. 8E). This observation would suggest that the combination of CD36 expression with intracellular lipid quantification allows for a more sensitive means of monitoring adipocyte differentiation.

Our data suggest that BDP is a sensitive indicator of the overall lipid content of cells. However, this increased sensitivity may result in the overestimation of changes in lipid content associated with adipogenesis. It is therefore important to normalize the data to a corresponding noninduced control when BDP is used as an indicator of adipogenesis-associated lipid content. The neutral core of lipid droplets is surrounded by an outer layer of amphipathic lipids, such as phospholipids and cholesterol (27, 57). In this study, we observed that adipocytes emit deep-red fluorescence, when stained with NR, as they mature. Based on the observation reported by Greenspan and Fowler (51) indicating that NR emits fluorescence in the deep-red fluorescence spectrum when dissolved in amphipathic lipids, we postulate that the observed emission of deep-red fluorescence might be due to the higher levels of amphipathic lipids that surround larger lipid droplets. We therefore postulate that the observed increase in deep-red fluorescence emission serves as an indicator of the presence of larger lipid droplets within adipocytes, and thus is an indicator of adipocyte maturation. Therefore, the unique emission spectrum of NR provides an opportunity to detect a more mature stage of adipocyte differentiation. Our results suggest that simultaneous staining with NR and CD36 allows for the identification of an even more mature adipocyte phenotype (CD36^high^/NR^FL2+/FL5+^) that displays an increased level of cellular complexity (Fig. 10). However, we recognize that quantification of the ratio of neutral versus amphipathic lipids in the various adipocyte subpopulations should be performed in future studies to confirm this hypothesis.

The differentiation process from mesenchymal stem cells to mature adipocytes is not synchronized, and various cell populations that are at different stages of differentiation are present at a given time point. To date, these intermediate cell populations have been poorly described, mainly due to lack of the necessary tools required to distinguish between the different populations, and this is therefore a limiting factor in fully understanding the different stages of adipogenesis (23). Currently two main populations are studied in the adipose differentiation process, i.e., preadipocytes and mature adipocytes. At a cellular level, investigators distinguish between these two cell populations based on cellular complexity and lipid content. Preadipocytes do have a lower degree of cellular complexity and contain less total lipid than mature adipocytes that display an increased level of cellular complexity and contain larger intracellular lipid droplets. Our data suggest that adipocytes can be classified into various subpopulations according to their CD36 expression profiles and intracellular neutral lipid content. In general, BDP seems to be more sensitive in detecting changes in intracellular lipid content, but NR’s unique fluorescence emission spectrum has the potential to allow for the identification of a more mature adipocyte phenotype.

Various investigators have shown that preadipocytes and mature adipocytes differ in their physiological function, levels of protein expression, etc. (11, 12, 19). It has also been shown that inflammation influences the ratio of preadipocytes to mature adipocytes in adipose tissue. However, the role and magnitude of intermediate adipocyte populations in inflammatory conditions is largely unknown. In this study, we provide a means of identifying and studying intermediate adipocyte populations. Our data clearly show that it is possible to monitor the distribution of various adipocyte subpopulations when several flow cytometric parameters and gene expression studies are combined. Therefore, the proposed model may contribute to a better understanding of the role of various intermediate phenotypes in the adipocyte differentiation process, both in physiological and pathological settings.

## Supplementary Material

Supplemental Data
