# Supplementary material for: Novel flow cytometric approach for the detection of adipocyte subpopulations during adipogenesis
Source: J Lipid Res. 2016 Apr;57(4):729–42. doi: 10.1194/jlr.D065664 (PMC4808761; doi:10.1194/jlr.D065664)
Supplement: Supplemental Data [file supp_57_4_729__index.html]

Novel flow cytometric approach for the detection of adipocyte sub-populations during adipogenesis — Novel flow cytometric approach for the detection of adipocyte subpopulations during adipogenesis — Supplemental Data 

# Novel flow cytometric approach for the detection of adipocyte subpopulations during adipogenesis

## Supplemental Data

- Supplemental Material - Durandt et al\_Noval flow cytometric approach (.pdf, 866 KB) - Supportive material to explain certain principles and data mentioned in text in more detail
