# Supplementary material for: Novel flow cytometric approach for the detection of adipocyte subpopulations during adipogenesis
Source: J Lipid Res. 2016 Apr;57(4):729–42. doi: 10.1194/jlr.D065664 (PMC4808761; doi:10.1194/jlr.D065664)
Supplement: Supplemental Data [file 10.1194_D065664_jlr.D065664-1.pdf]

SUPPLEMENTARY DATA

Figure 1

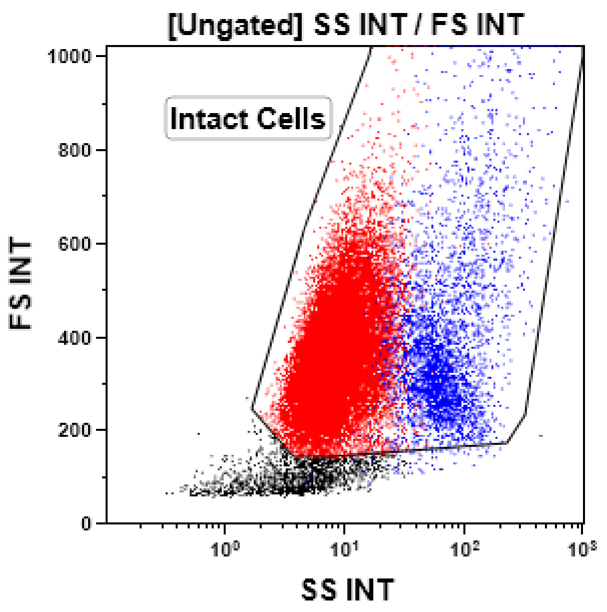

Supplementary Figure 1: A Forward Scatter (FS) Lin vs Side Scatter (SS) Log dot plot to indicate the gating followed to identify intact cells during flow cytometric analyses. Culture A041214 P5 was used as example. Analysis was performed 21 days post induction. Intact cells (intermediate/high forward scatter; red & blue populations) were distinguished from cellular debris (low forward scatter; black population). An increase in intracellular lipid droplets resulted in an increase in cellular complexity (increase in side scatter) as indicated by the blue cell population.

Figure 2

A: Day 0 – Not Induced

B: Day 21 – Not Induced

C: Day 21- Induced

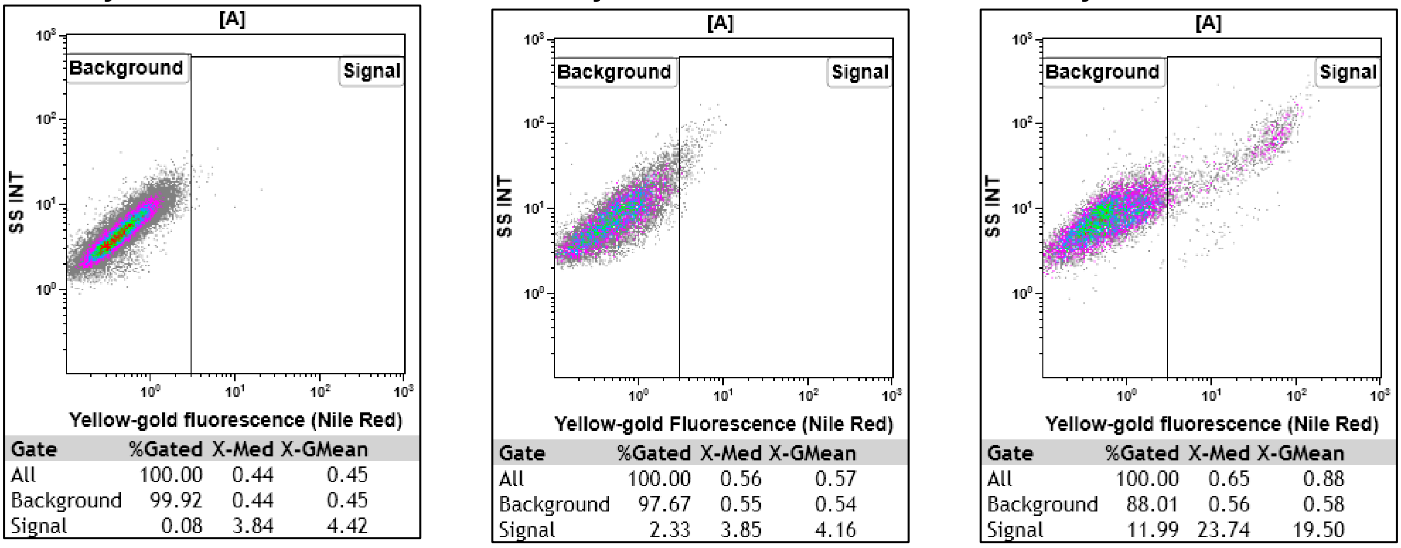

Supplementary Figure 2: Representative flow cytometry density plots (SS Log vs FL2) to indicate the fluorescence intensities observed for ASCs after staining with Nile Red at A: day 0 (prior induction), B: day 21 (not induced) and C: day 21 (induced). X-Med refers to the relative median fluorescence intensity observed for all yellow-gold fluorescent events represented in either the “Background” or the “Signal” region. X-GMean refers to the relative geometric mean fluorescence intensity observed for all yellow-gold fluorescent events represented in either the “Background” or the “Signal” region. The “Signal” region represents all cells that emit fluorescence higher/brighter than the relative background fluorescence observed. A similar strategy was used to determine the signal (FL1): background ratio for Bodipy 493/503.

The formula applied by the software to calculate the median fluorescent intensity of cells present in a specific region of interest (directly quoted from Instructions for Use manual, Kaluza Flow Cytometry Software, Beckman Coulter (Miami, USA)) is:

“Median of the values of the events in the input gate (region of interest). Kaluza Analysis computes the frequency histogram of the events to generate this statistic. The frequency histogram has 1024 bins.”

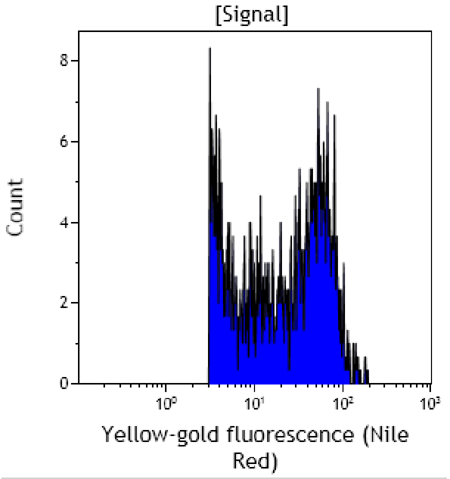

Gate	Number	%Gated	X-Med	X-GMean
All	1 201	100.00	23.74	19.50

An example of the frequency histogram created for the “Signal” region (Supplementary Figure 2C)

$$b = i \left| \sum_{i=1}^{j} C_i > \frac{n}{2} \right.$$

$[j \leq n, C_i = Count\ in\ bin\ i, n = Total\ number\ of\ events\ in\ input\ gate]$

The median m can then be computed using the following steps.

1. $A = \frac{\sum_{i=1}^{b-1} x_i}{n} * 100$
2. $B = 50 - A$
3. $D = \frac{k_b}{n} * 100$
4. $E = \frac{B}{D}$
5. $m = b + E$

In step 3, k_b is the count in bin b.

Figure 3

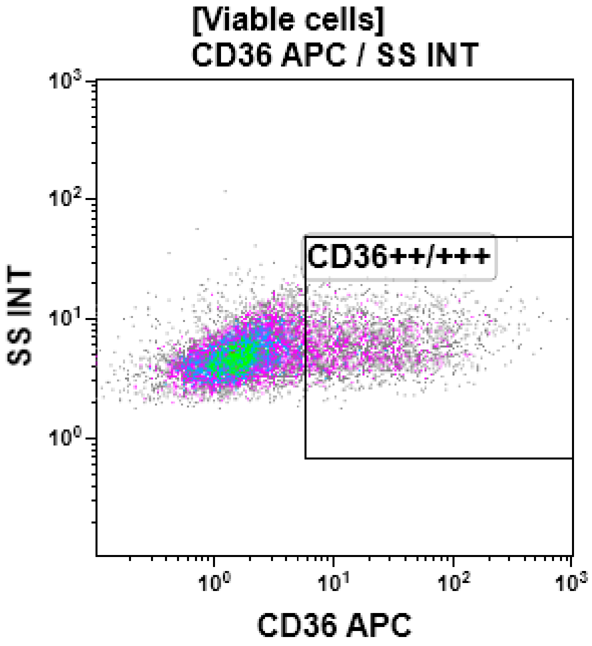

Supplementary Figure 3: A flow cytometry density plot (SS Log vs CD36 APC) to indicate the two levels of CD36 expression observed. The majority of cells expressed CD36 at low levels, while a sub-population of cells expressed CD36 at intermediate/high levels.

Figure 4

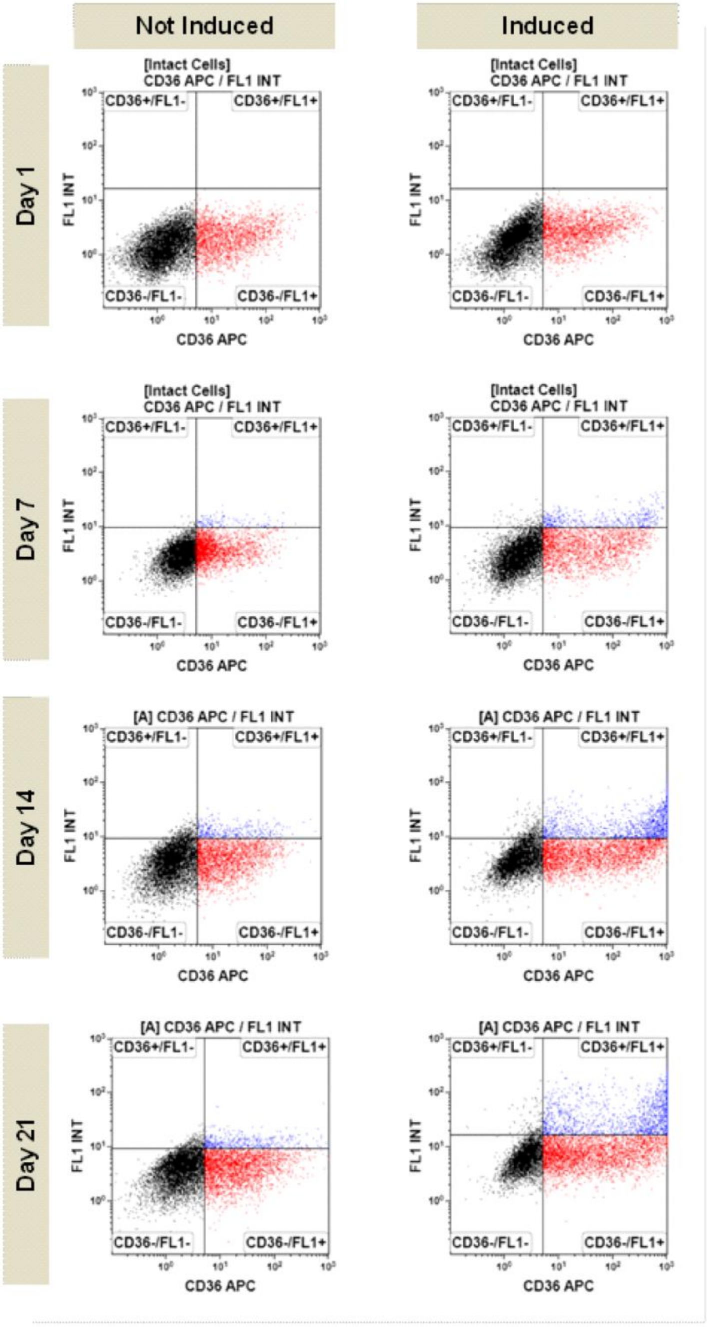

Not Induced
Induced
Day 1
Day 7
Day 14
Day 21
[Intact Cells]
CD36 APC / FL1 INT
[A] CD36 APC / FL1 INT
CD36+/FL1-
CD36+/FL1+
CD36-/FL1-
CD36-/FL1+
FL1 INT
CD36 APC

Supplementary Figure 4: An example of flow cytometric data obtained at the various time points for a specific culture (A180813 P15F1) after the cells were simultaneously stained with Bodipy 493/503 and CD36-APC. Results indicate that adipocyte differentiation is initially associated with an increase in the level of expression of CD36 (day 1 & day 7; induced culture). An increase in lipid accumulation (FL1+) was only detected at high levels of CD36 expression (day 14 & day 21; induced culture).

Figure 5

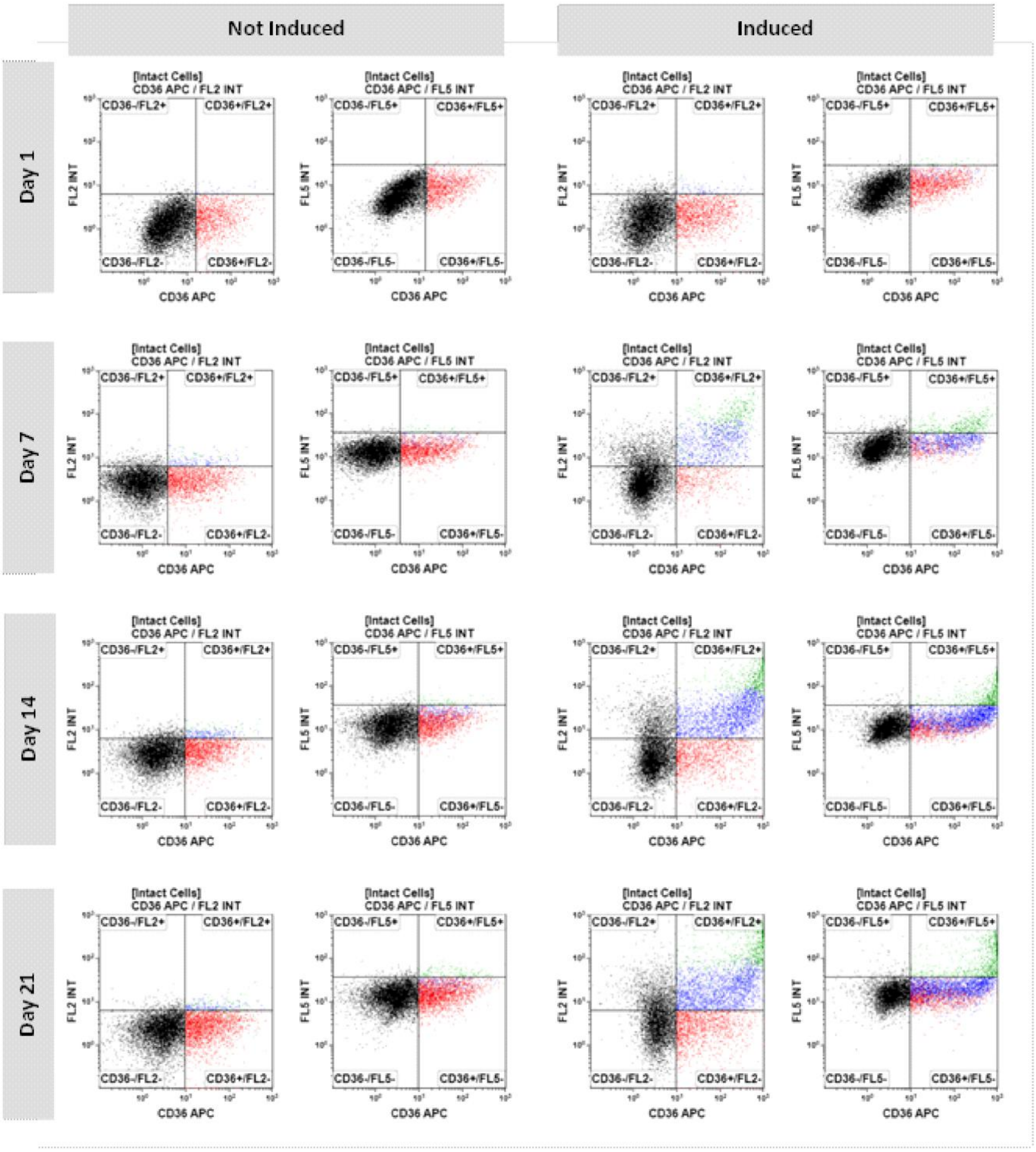
Not Induced
Induced
Day 1
Day 7
Day 14
Day 21
[Intact Cells]
CD36 APC / FL2 INT
CD36 APC / FL5 INT
CD36-/FL2+
CD36+/FL2+
CD36-/FL2-
CD36+/FL2-
CD36-/FL5+
CD36+/FL5+
CD36-/FL5-
CD36+/FL5-
FL2 INT
FL5 INT
CD36 APC

Supplementary Figure 5: An example of flow cytometric data obtained at the various time points for a specific culture (A180813) after cells were simultaneously stained with Nile Red and CD36-APC. Results indicate that adipocyte differentiation is initially associated with an increase in the level of expression of CD36 as well as yellow-gold fluorescence emission (FL2+) (day 1 & day 7; induced culture). An increase in deep-red fluorescence (FL5+) is only detected at high levels of CD36 and yellow-gold fluorescence (day 14 & day 21; induced culture).
